# Long noncoding RNAs regulated spermatogenesis in varicocele‐induced spermatogenic dysfunction

**DOI:** 10.1111/cpr.13220

**Published:** 2022-03-17

**Authors:** Shangren Wang, Jiaqi Kang, Yuxuan Song, Aiqiao Zhang, Yang Pan, Zhexin Zhang, Yuezheng Li, Shuai Niu, Li Liu, Xiaoqiang Liu

**Affiliations:** ^1^ Department of Urology Tianjin Medical University General Hospital Tianjin China; ^2^ Department of Urology Peking University People's Hospital Beijing China; ^3^ Biomedical Pioneering Innovation Center (BIOPIC), School of Life Sciences Peking University Beijing China; ^4^ Department of Neonatology First Teaching Hospital of Tianjin University of Traditional Chinese Medicine Tianjin China; ^5^ Department of Neonatology National Clinical Research Center for Chinese Medicine Acupuncture and Moxibustion Tianjin China

## Abstract

**Objectives:**

To evaluate the expression, potential functions and mechanisms of long noncoding RNAs (lncRNAs) in the pathogenesis of varicocele (VC)‐induced spermatogenic dysfunction.

**Materials and Methods:**

We established a rat model with left experimental VC and divided rats into the sham group, the VC group, and the surgical treatment group (each group, *n* = 10). Haematoxylin and eosin (HE) staining and sperm quality were analysed to evaluate spermatogenesis function. LncRNA expression profiles were analysed using lncRNA‐Seq (each group *n* = 3) and validated using quantitative real‐time polymerase chain reaction (each group *n* = 10). Correlation analysis and gene target miRNA prediction were used to construct competing endogenous RNA network. The regulated signalling pathway and spermatogenic dysfunction of differentially expressed lncRNAs (DE lncRNAs) were validated by Western blot.

**Results:**

HE detection and sperm quality analysis showed that VC could induce spermatogenic dysfunction. Eight lncRNAs were upregulated and three lncRNAs were downregulated in the VC group compared with the sham group and surgical treatment group. The lncRNA of *NONRATG002949.2*, *NONRATG001060.2*, *NONRATG013271.2*, *NONRATG022879.2*, *NONRATG023424.2*, *NONRATG005667.2* and *NONRATG010686.2* were significantly negatively related to sperm quality, while *NONRATG027523.1*, *NONRATG017183.2* and *NONRATG023747.2* were positively related to sperm quality. The lncRNAs promote spermatogenic cell apoptosis and inhibit spermatogonia and spermatocyte proliferation and meiotic spermatocytes by regulating the PI3K–Akt signalling pathway.

**Conclusion:**

DE lncRNAs may be potential biomarkers for predicting the risk of spermatogenic dysfunction in VC and the effect of surgical repair. These DE lncRNAs promote spermatogenic dysfunction by regulating the PI3K–Akt signalling pathway.

## Graphical abstract

The lncRNAs may be potential novel biomarkers for predicting the risk of spermatogenic dysfunction in VC and the effect of surgical repair. These lncRNAs promote spermatogenic cell apoptosis and inhibit spermatogonia and spermatocyte proliferation and meiotic spermatocytes by regulating the PI3K–Akt signalling pathway and other other mechanisms.

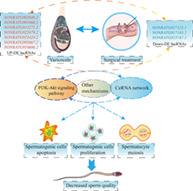

**Abbreviations**
Akt: protein kinase B
Bax: Bcl‐2‐associated X protein
Bcl‐2: B‐cell lymphoma‐2
PCNA: proliferating cell nuclear antigen
PI3K: phosphatidylinositol 3‐kinase
PLZF: promyelocytic leukaemia zinc finger protein
REC8: recombination 8
STRA8: retinoic acid gene 8
SYCP3: synaptonemal complex protein 3

## 1 INTRODUCTION

Varicocele (VC) is a common disease in male infertility in which the internal spermatic vein malformation is twisted, dilated, and elongated.^1^ The prevalence of VC in the general population is approximately 15%, and in infertile males, it is approximately 35%.^2^, ^3^ VC is a common aetiology of male infertility. Studies have shown that VC plays a role in decreased testicular function, leading to spermatogenic dysfunction and diminished testosterone levels.^4^, ^5^ Some mechanisms may contribute to VC‐induced spermatogenic dysfunction, including the ionic imbalance, high testicular temperature, neuroendocrine system dysfunction, hypoxia, chronic oxidative and disruption of the blood–testis barrier.^6^, ^7^, ^8^, ^9^, ^10^ Those chronic stress may reduce the function of spermatogenesis and damage the structure of spermatogenic cells such as DNA, RNA, lipids and proteins resulting in poor sperm quality.^10^ However, the exact mechanisms remain unclear and require more research.

Long noncoding RNAs (lncRNAs), a kind of noncoding RNA (ncRNA), are more than 200 nucleotides in length and lack functional protein‐coding ability.^11^ Some studies have shown that lncRNAs play an essential role in the adjustment of gene expression and have broad functions in many critical biological processes, such as genomic imprinting, differentiation, apoptosis, nuclear organization, alternative splicing and nuclear import.^11^ LncRNAs participate in various disease processes, such as diabetes, cardiovascular disease and cancers.^12^, ^13^ Wen et al. showed that testis‐specific lncRNAs play an important role in late *Drosophila* spermatogenesis.^14^ Sanei‐Ataabadi et al. found that oxidative‐related lncRNAs are related to VC‐connected male sterility.^15^ Although many lncRNAs exist in spermatogenesis, their expression and function in spermatogenic dysfunction induced by VC remain to be studied.

To understand the expression and function of lncRNAs in VC‐induced spermatogenic dysfunction, in the present study, we performed RNA‐seq to profile lncRNA expression in VC rats, and the results were validated for expression in the testis and relationships with sperm quality. The findings will contribute to understanding these mechanisms of spermatogenic dysfunction caused by VC and identifying new biomarkers for the diagnosis and treatment of VC‐induced spermatogenic dysfunction.

## 2 MATERIALS AND METHODS

### 2.1 Animals

Thirty male Sprague Dawley rats aged 6–7 weeks were obtained from the Institute of Radiation Medicine, Chinese Academy of Medical Sciences. After 7 days of adaptive feeding, the rats were randomized into three groups: sham group (*n* = 10), VC group (*n* = 10), and surgical treatment group (*n* = 10). The research was supported by the Ethics Committee of Tianjin Medical University General Hospital (Approval No. IRB2021‐DW‐51).

Based on Turner's previously published surgical protocol, we built a left experimental VC rat model.^16^ The sham group received a similar treatment without left renal vein obstruction. The VC rat models tested the sperm vein diameter at 8 weeks after modelling. Compared with the sham group, a more than twofold increase in the outer diameter of the left spermatic vein is considered a successful VC model. Meanwhile, the surgical treatment group received varicocelectomy at 8 weeks after modelling. The steps were as follows: open the abdomen layer by layer, separate the spermatic vein, ligate the vein with 4–0 silk thread, test the outer diameter of the left spermatic vein after ligating, and finally, close the abdomen layer by layer. Samples were obtained for next analysis at 4 weeks after varicocelectomy.

### 2.2 Semen analysis

The left caudal epididymis was minced in phosphate‐buffered saline, and the sperm were released after incubation for 5 min at 37°C. The sperm count and sperm motility, including progressive (PR) and nonprogressive (NP) motility, were analysed using a computer‐aided analysis system (Weili). Total motility is defined as the percentage of PR and NR sperm motility.

### 2.3 Histological examination

Fresh testicular tissues were immobilized with 4% formalin for 1 day, dehydrated in the presence of increased ethanol concentrations, and embedded in paraffin for sectioning. The sections were stained with haematoxylin and eosin (HE) dye and observed under a light microscope.

### 2.4 Total RNA extraction and lncRNA sequencing

We extracted total RNA of fresh testicular tissues with TRIzol (Invitrogen) and qualified it with an Agilent 2100 Bioanalyzer (Agilent Technologies), a NanoDrop spectrophotometer (Thermo Fisher Scientific) and 1% (wt/vol) agarose gels. Three rats from each group were randomly selected for lncRNA sequencing. High‐throughput sequencing and subsequent data analysis were implemented by GENESKY Biotechnologies Inc. using the standard Illumina HiSeq 2500 platform.

### 2.5 Identification of differentially expressed lncRNAs

The edgeR package^17^ was applied to identify the differentially expressed lncRNAs (DE lncRNAs) using the standard of |log(fold change)| > 1 and adjusted *p* < 0.05. We analysed the comparisons between the VC group versus the sham group and the VC group versus the surgical treatment group to generate DE lncRNAs. DE lncRNAs were visualized by heatmaps and volcano maps drawn by the heatmap and ggplot2 packages. Overlapping dysregulated DE lncRNAs were screened for comparison between the two groups by Venny2.1 (https://bioinfogp.cnb.csic.es/tools/venny/index.html).

### 2.6 Construction of lncRNA–miRNA–mRNA network

We predicted interactions between lncRNAs and miRNAs by miRanda software with a perfect seed match. Full‐length sequences of lncRNA and miRNA were selected. miRNA–mRNA interactions were identified by combining RNAhybird and miRanda software. Then, we screened pairs of positive correlations between the expression of lncRNAs and mRNAs, and the lncRNA–miRNA–mRNA competing endogenous RNA (ceRNA) network was obtained and visualized with Cytoscape v3.7.1 software.^18^

### 2.7 Protein–protein interaction network

We constructed protein–protein interaction (PPI) networks based on predicted target genes using the interactive gene retrieval tools (STRING) database^19^ and visualized them through Cytoscape v3.7.1.^19^ The cytoHubba plug‐in was used to rank target genes and identify hub genes.

### 2.8 Functional enrichment analysis

To investigate the biological function of DE lncRNAs, we performed Gene Ontology (GO) and Kyoto Encyclopedia of Genes and Genomes (KEGG) pathway enrichment analyses, including biological process, molecular function and cellular component analyses, for target genes and hub genes by Metascape.^20^ Terms with *p* < 0.05 were considered statistically significant.

### 2.9 Validation by quantitative real‐time polymerase chain reaction

We extracted total RNA from testicular tissue samples using TRIzol reagent (Invitrogen) according to the manufacturer's instructions. Then, first‐strand cDNA Synthesis Super Mix for qPCR (Yeasen) was used to amplify the cDNA. Subsequently, a SYBR Green PCR kit (Yeasen) was used for quantitative real‐time polymerase chain reaction (RT‐PCR) with a cDNA template on a real‐time detector (MA‐6000; Molarray). Quantifications were normalized to GAPDH and were analysed using the 2^−ΔΔ*C*t^ method. Table 1 shows the primer sequences.

**TABLE 1:** Primer pairs used in this study

lncRNAs	Primer sequence(5′–3′)	
NONRATG001060	Forward	TCCAGTTAAGCAGTTAAGCCTTCCTC
	Reverse	GTCCTGAGCCTGACTCTGTACCA
NONRATG002949	Forward	AGAAGCCAGCAGAGTGACGAGAA
	Reverse	TCACATCAAATGAGGGCTGTAGACAA
NONRATG005667	Forward	ATGACAGCGTGGTTGACAGTGTT
	Reverse	CATTCCTGGTTCTGACGAGTAGCC
NONRATG007482	Forward	TCCTAGCCTGAATAACCGTGAGCAA
	Reverse	ACTGAGAGATTGTCCACCACCCTTC
NONRATG010686	Forward	CATTACCTGGCTTCAATCAAGGCATT
	Reverse	GCAAGACCCAAGAATTTCAGTTCCAA
NONRATG013271	Forward	TTGCCTCAGGAAGAACAGGTAGAAGA
	Reverse	CAGGAGATTCGCCACATCGTCAG
NONRATG017183	Forward	TCTTCTTCTTCCTTCCTGCCTTCCT
	Reverse	ACACGCTTTAATCCCAACACTGAGAT
NONRATG022879	Forward	TTCTGCCTTGAAACCGACCAAATACT
	Reverse	ACTCAGGAAGACTGCTACTGGAAGAT
NONRATG023424	Forward	CTGCTTGCTGTCTGAGTTCTCTACC
	Reverse	CAATTCTCCTGCCTCCACCTTCC
NONRATG023747	Forward	ATCCTCACAACAGTGGTGGCTCTA
	Reverse	GACTCGGTATCTGCTTCCGCTTAG
NONRATG027523	Forward	AGGGAGGTGGTATTCAGGACTTTGG
	Reverse	AGTGGCTGGTAACTAGGAAGAGGAAG
GAPDH	Forward	GGCAAGTTCAACGGCACAG
	Reverse	CGCCAGTAGACTCCACGACA

### 2.10 Apoptosis tests

Terminal deoxynucleotidyl transferase nick end labeling (TUNEL) staining was performed using the TUNEL Assay Kit (KGA7073; 7Sea Boitech) according to the manufacturer's instructions. Pictures were taken under a microscope for green fluorescence generated by TUNEL‐positive cells and red fluorescence generated by total DNA. TUNEL positivity was calculated by dividing the total number of TUNEL‐positive cells by the number of nuclei.

### 2.11 Western blot

Testicular tissues of rats were prepared using radioimmunoprecipitation assay buffer containing protease inhibitor. BCA (Solarbio) was used to test protein concentrations. The protein was separated by electrophoresis and transferred to membranes. The following primary antibodies were used for incubation with membranes: PI3K (1:1000; Abcam), Akt (1:1000; Affinity Biosciences), p‐Akt (1:1000; Affinity Biosciences), caspase‐9 (1:1000; Affinity Biosciences), Bcl‐2 (1:1000; Affinity Biosciences), Bax (1:1000; Affinity Biosciences), PCNA (1:1000; Affinity Biosciences), PLZF (1:1000; Affinity Biosciences), REC8 (1:1000; Bioss), STRA8 (1:1000; Affinity Biosciences), SYCP3 (1:1000; Affinity Biosciences), GAPDH (1:1000). Then, the membranes were incubated with a secondary antibody (Bioss). Subsequent visualization with a chemiluminescent imaging system was performed (KPL).

### 2.12 Statistical analysis

Data were represented by the mean ± *SD*. Unpaired Student's *t*‐test was used to compare parameter data between groups. The Mann–Whitney rank‐sum test was used for nonparametric data. We carried out a Pearson correlation analysis between DE lncRNA expression and semen parameters. We carried out all statistical analyses using GraphPad Prism 8.0 (GraphPad Software). A result of *p* < 0.05 was regarded with statistical significance.

## 3 RESULTS

**FIGURE 1:**
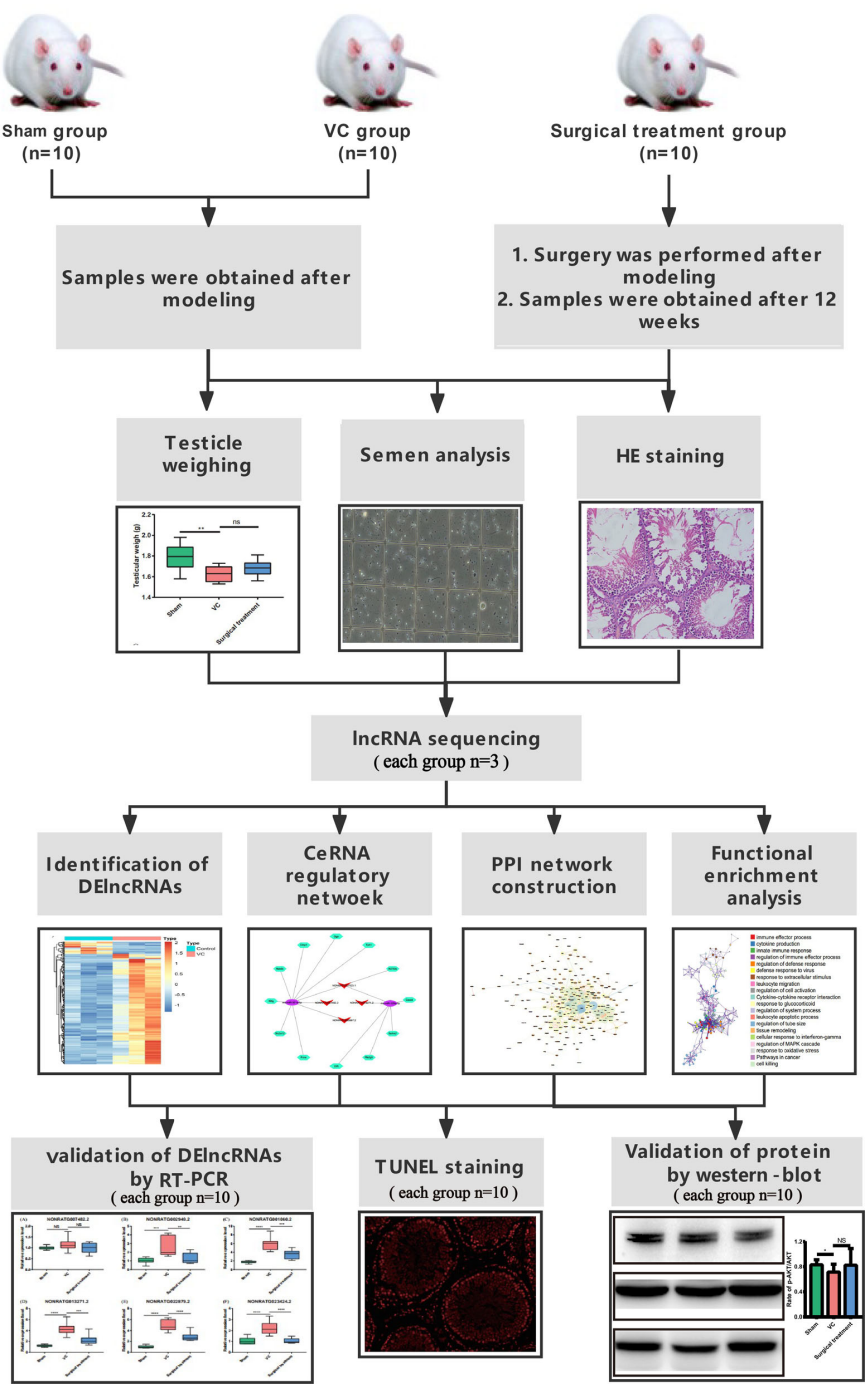
Workflow. CeRNA, competing endogenous RNAs; DE lncRNAs, differentially expressed lncRNAs; HE, haematoxylin and eosin; PPI, protein–protein interaction; RT‐PCR, quantitative real‐time polymerase chain reaction; VC, varicocele

**FIGURE 2:**
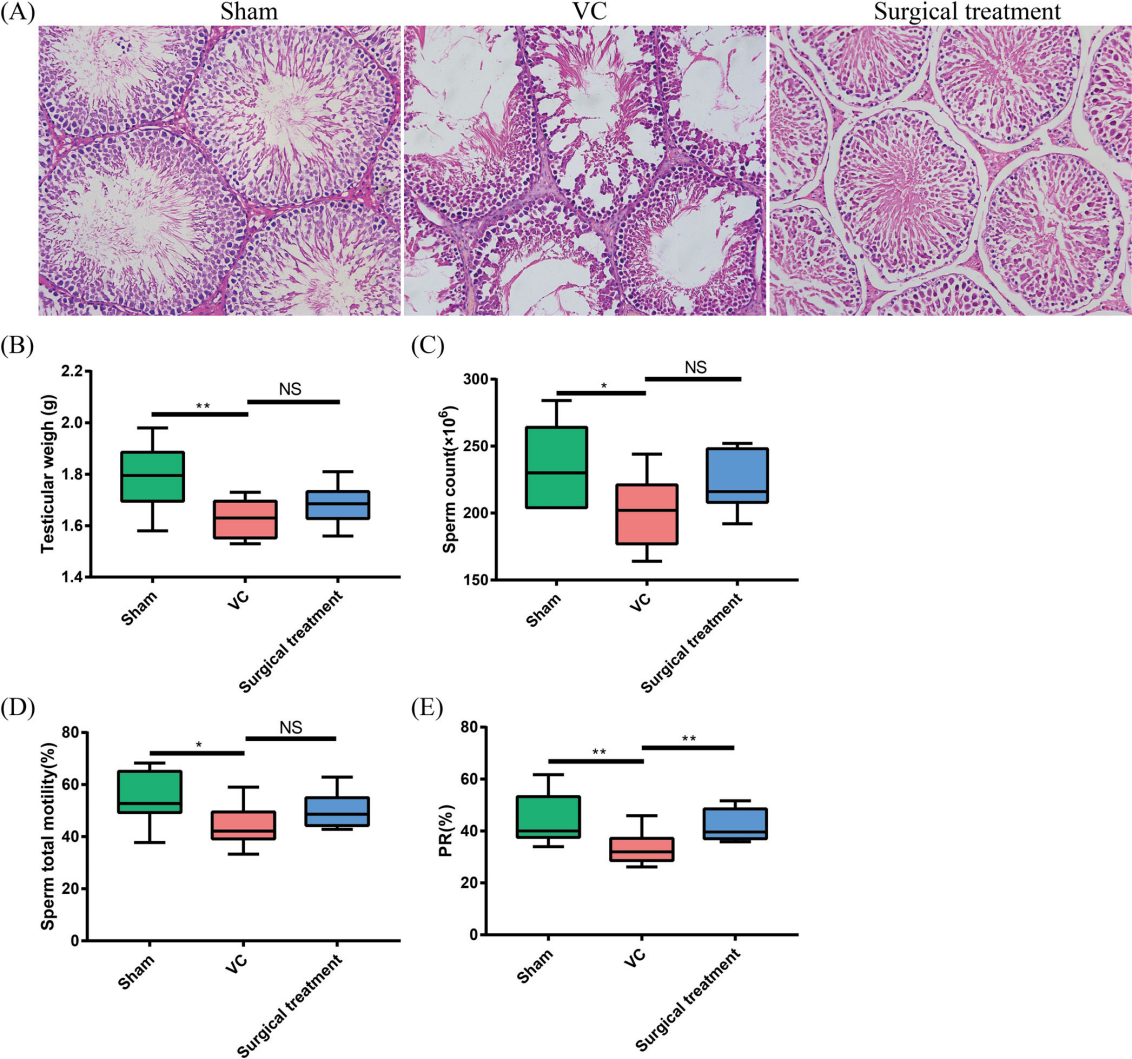
HE staining of rat testicular tissues, basic parameters and semen analysis in rats. (A) Representative HE staining image of rat testicular tissues (×200). (B) Testicular weight of lift, (C) sperm count, (D) sperm total motility, and (D) PR% of sperm. *p <0.05; **p <0.01; HE, haematoxylin and eosin; NS, not significant; PR, sperm progressive motility; surgical treatment, surgical treatment group; sham: sham group; VC, varicocele group

The research flow chart is summarized in Figure 1. As showed in Table 2, 8 weeks after modelling, VC model rats showed significant dilation of the spermatic vein and body weights among the three groups were not significantly different. The weight of the left testis in the sham group was higher than that in the VC group (*p* < 0.01, Figure 2B) and was not significantly different between the VC group and the surgical treatment group (*p* > 0.05, Figure 2B). The sperm count, total motility and PR motility of rats in the sham group were apparently higher than those in the VC group (Figure 2C–E). The sperm count and total motility in rats after surgical treatment did not increase significantly (Figure 2C,D), but the number of PR motility sperm increased significantly compared with the VC group **(** *p* < 0.01, Figure 2E).

**TABLE 2:** Basic parameters and semen analysis in rats

Group	Sham group	VC group	Surgical treatment group	*p* Value^a^	*p* Value^b^
Body weight at 8 weeks (g)	361.10 ± 2.09	362.90 ± 1.43	361.70 ± 1.47	0.4866	0.5660
Lift testicular weight (g)	1.79 ± 0.04	1.63 ± 0.02	1.68 ± 0.02	0.0036	0.1372
Right testicular weight (g)	1.75 ± 0.04	1.65 ± 0.03	1.67 ± 0.03	0.0602	0.5643
Left sperm vein diameter (mm)	0.372 ± 0.01	1.597 ± 0.02	0.602 ± 0.02	<0.0001	<0.0001
Sperm concentration (×10^6^)	234.4 ± 9.58	201.2 ± 8.39	222.8 ± 6.90	0.0178	0.0623
Progressive motility (PR%)	44.91 ± 3.10	33.39 ± 1.96	42.30 ± 1.911	0.0057	0.0044
Total motility (PR + NP%)	54.83 ± 3.10	43.76 ± 2.36	50.29 ± 2.07	0.0108	0.0519

Abbreviations: NP, nonprogressive; PR, progressive; VC, varicocele.

^a^ *p* Value for Sham group vs. VC group.

^b^ *p* Value for VC group vs. surgical treatment group.

### 3.1 HE staining of rat testicular tissues

As shown in Figure 2A, HE staining analysis further reflected that the counts of spermatogonia, spermatocytes and round spermatids in the seminiferous tubules of the VC group were significantly reduced, while those in the surgical treatment group were significantly increased.

### 3.2 Identification of the coexpression of DE lncRNAs in the VC group

**FIGURE 3:**
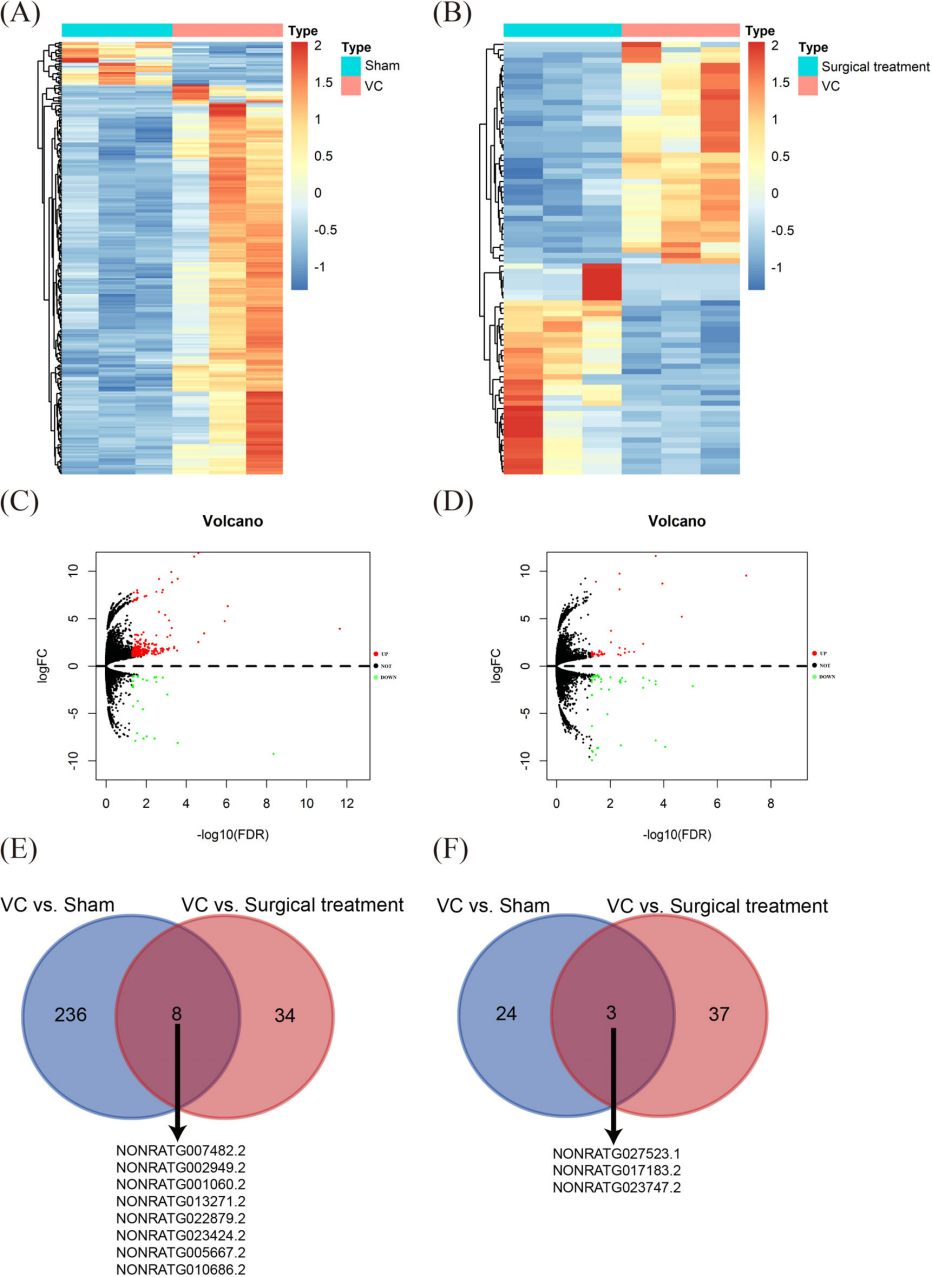
The expression profiles of lncRNAs in VC rats. (A) Heatmap for sham group versus VC group. (B) Heatmap for surgical treatment group versus VC group. (C) Volcano map for VC group versus sham group. (D) Volcano map for VC group versus surgical treatment group. (E) Venn diagram showing upregulation of eight DE lncRNAs among the surgical treatment group versus VC group and VC group versus sham group comparisons. (F) Venn diagram showing downregulation of three DE lncRNAs among the surgical treatment group vs. VC group and VC group vs. sham group comparisons comparisons. Down, downregulation; NOT, not significant; Sham, sham group; Surgical treatment, surgical treatment group; Up, upregulation; VC, varicocele group

To elucidate the mechanism of impaired sperm quality in the testes of VC rats in more detail, entire testis samples for lncRNA sequencing were collected. Compared with those in the sham group, 244 upregulated lncRNAs and 27 downregulated DE lncRNAs were detected in the VC group (Figure 3A,C). In the comparison between the VC group and the surgical treatment group, we identified 82 DE lncRNAs, including 42 upregulated and 40 downregulated DE lncRNAs (Figure 3B,D). Venn diagram analysis showed that 11 DE lncRNAs overlapped between the two comparisons (Figure 3E,F). Specifically, eight DE lncRNAs were upregulated and three DE lncRNAs were downregulated in the VC group (Table 3).

**TABLE 3:** Key lncRNAs in the differential expression analysis

lncRNAs	VC group vs. Sham group		VC group vs. Surgical treatment group	
	logFC	Adjusted *p* value	logFC	Adjusted *p* value
NONRATG007482.2	1.332	0.012	1.165	0.050
NONRATG002949.2	1.578	0.014	2.317	0.001
NONRATG001060.2	9.918	0.001	9.752	0.004
NONRATG013271.2	1.201	0.018	1.234	0.041
NONRATG022879.2	1.293	0.003	1.160	0.029
NONRATG023424.2	1.650	0.003	1.798	0.003
NONRATG005667.2	1.347	0.024	1.450	0.021
NONRATG010686.2	1.363	0.028	1.484	0.048
NONRATG027523.1	−9.253	<0.001	−8.369	0.004
NONRATG017183.2	−7.654	0.004	−8.541	<0.001
NONRATG023747.2	−1.505	0.010	−2.128	<0.001

Abbreviation: VC, varicocele.

### 3.3 The regulatory lncRNA–miRNA–mRNA network

**FIGURE 4:**
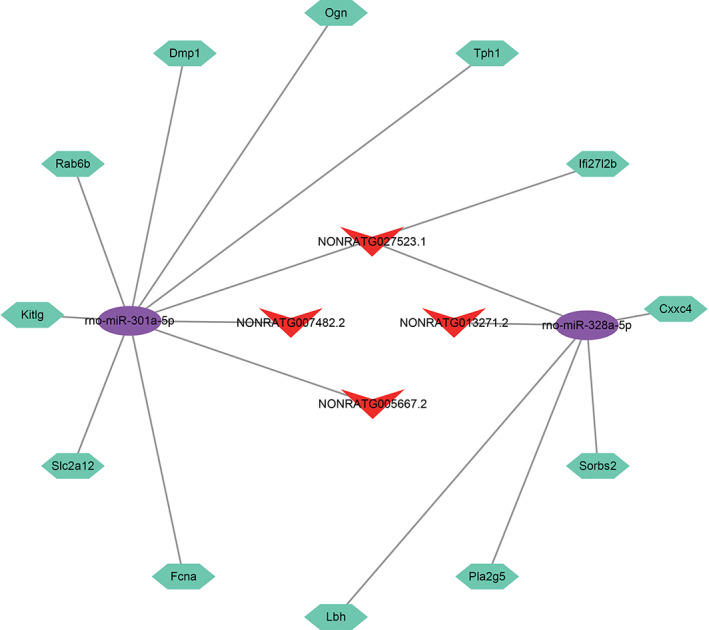
The regulatory lncRNA–miRNA–mRNA network. Red: lncRNAs, purple: miRNAs, cyan‐blue: targeted genes

LncRNAs can act as miRNA sponges via ceRNA networks to regulate miRNA‐targeted gene expression.^21^ Mo‐miR‐301a‐5p and mo‐miR‐328a‐5p, which can potentially bind to four DE lncRNAs, were identified and are shown in Figure 4. The predicted mRNAs were selected as potential target genes in at least two of the databases used (RNAhybird and miRanda). Finally, lncRNA–miRNA–mRNA regulatory networks were constructed based on 4 lncRNAs, 2 miRNAs, and 12 mRNAs (Figure 4).

### 3.4 Functional enrichment analysis of target genes

**FIGURE 5:**
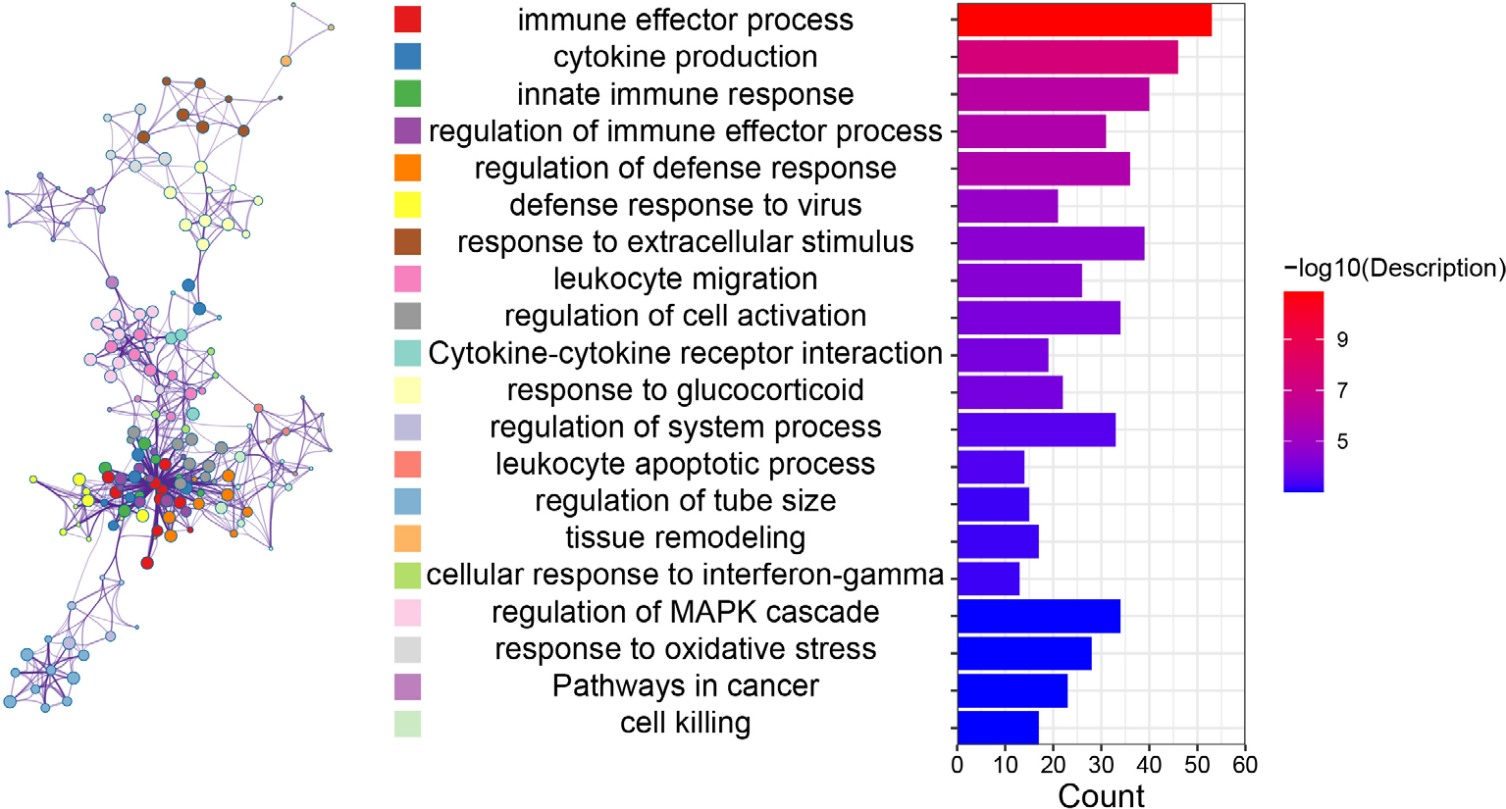
Functional enrichment analysis of target genes

**FIGURE 6:**
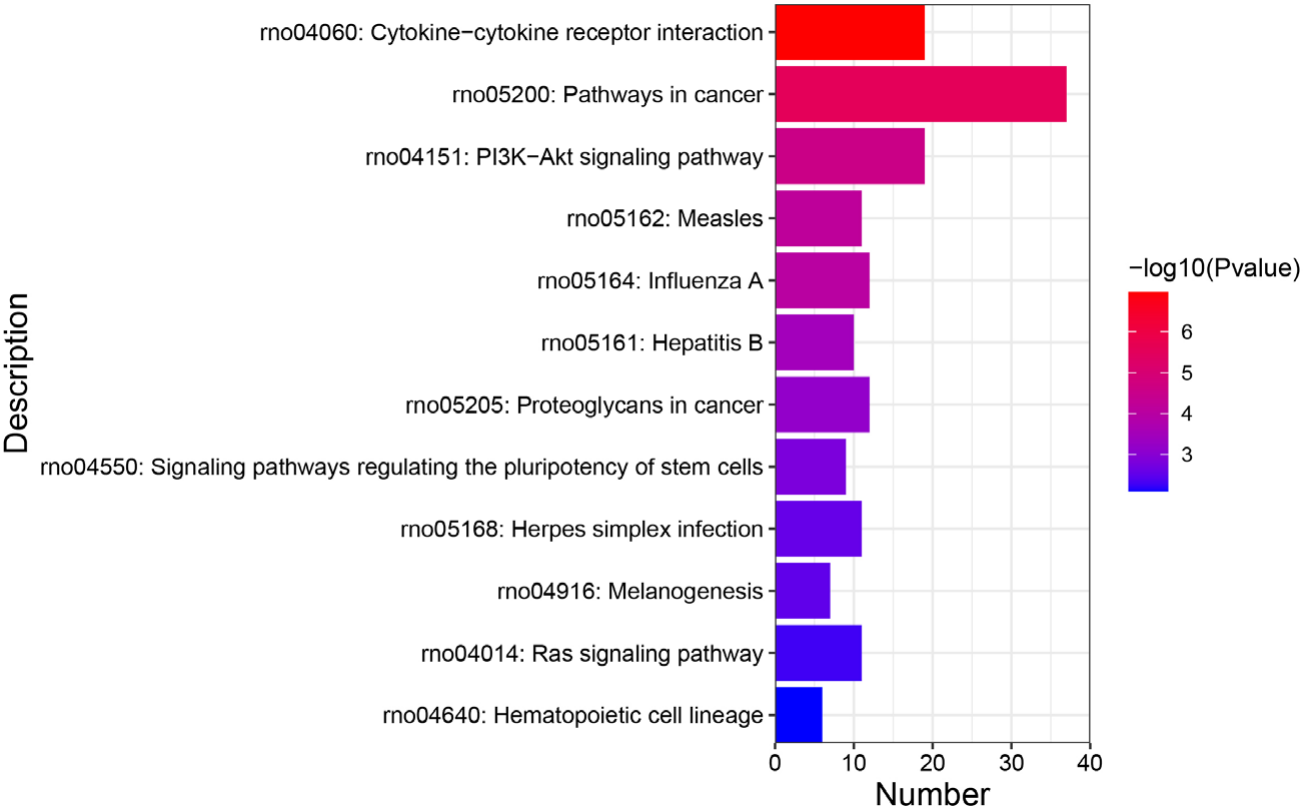
Kyoto Encyclopedia of Genes and Genomes (KEGG) pathway functional enrichment analysis

For further research, the biological processes and pathways based on the established ceRNA network were explored. The DE lncRNAs were closely related to inflammation or immune‐associated biological processes, apoptosis and oxidative stress, such as immune effector processes, cytokine production, innate immune response, defensive reaction regulation, leukocyte apoptotic process and response to oxidative stress (Figure 5 and Table 4). Similarly, several KEGG pathways were identified, including cytokine–cytokine receptor interactions, pathways in cancer and the PI3K–Akt signalling pathway (Figure 6 and Table 5). Overall, these results suggest that the DE lncRNAs are correlated with the behaviour of VC.

**TABLE 4:** Functional enrichment analysis of target genes

Category	Term	Description	FDR	InTerm_InList
GO biological processes	GO:0002252	Immune effector process	1.38E−11	53/764
GO biological processes	GO:0001816	Cytokine production	2.95E−08	46/737
GO biological processes	GO:0045087	Innate immune response	8.08E−07	40/659
GO biological processes	GO:0002697	Regulation of immune effector process	1.74E−06	31/434
GO biological processes	GO:0031347	Regulation of defence response	1.78E−06	36/571
GO biological processes	GO:0051607	Defence response to virus	1.45E−05	21/236
GO biological processes	GO:0009991	Response to extracellular stimulus	2.99E−05	39/746
GO biological processes	GO:0050900	Leukocyte migration	3.66E−05	26/378
GO biological processes	GO:0050865	Regulation of cell activation	6.60E−05	34/619
KEGG pathway	ko04060	Cytokine–cytokine receptor interaction	8.64E−05	19/226
GO biological processes	GO:0051384	Response to glucocorticoid	8.81E−05	22/300
GO biological processes	GO:0044057	Regulation of system process	2.96E−04	33/647
GO biological processes	GO:0071887	Leukocyte apoptotic process	3.43E−04	14/140
GO biological processes	GO:0035150	Regulation of tube size	5.46E−04	15/168
GO biological processes	GO:0048771	Tissue remodelling	5.53E−04	17/215
GO biological processes	GO:0071346	Cellular response to interferon‐gamma	5.53E−04	13/127
GO biological processes	GO:0043408	Regulation of MAPK cascade	9.64E−04	34/729
GO biological processes	GO:0006979	Response to oxidative stress	9.75E−04	28/538
KEGG pathway	rno05200	Pathways in cancer	9.80E−04	23/390
GO biological processes	GO:0001906	Cell killing	9.80E−04	17/229

**TABLE 5:** Kyoto Encyclopedia of Genes and Genomes (KEGG) pathway functional enrichment analysis

Term	Description	*p* Value	Symbols
rno05200	Pathways in cancer	2.82E−06	Hgf, Cxcl12, Fgfr2, Mitf, Stat1, Pdgfa, Pdgfra, Casp3, F2r, Rock2, Egln3, Fzd1, Kitlg, Gng11, Tgfb2, Rxrg, Gng10, Wnt2b, Wnt11, Fas, Wnt9b, Csf1r, Fos, Tlr4, Pxn, Mapk11, Wls, Osr1, Hnf1a, Jak3, Meis1, Edn1, Tnfrsf11b, Irs1, Rbp4, Hoxd4, Adm
rno05162	Measles	6.58E−05	Stat1, Jak3, Tlr4, Eif2ak2, Oas1a, Fas, Mx2, Fcgr2b, Irf7, Ddx58, Ifih1
rno05164	Influenza A	9.47E−05	Stat1, Tlr4, Eif2ak2, Rsad2, Oas1a, Fas, Mx2, Irf7, Ddx58, Il33, Ifih1, Mapk11
rno05161	Hepatitis B	3.45E−04	Stat1, Casp3, Tlr4, Tgfb2, Fas, Irf7, Ddx58, Fos, Ifih1, Creb5
rno05168	Herpes simplex infection	2.80E−03	RT1‐M3‐1, Tap1, Stat1, Casp3, Eif2ak2, Oas1a, Fas, Irf7, Ddx58, Fos, Ifih1
rno04060	Cytokine–cytokine receptor interaction	1.07E−07	Hgf, Il6r, Il9r, Cxcl12, Pdgfa, Pdgfra, Tnfrsf11b, Csf3, Ccl11, Kitlg, Tgfb2, Cx3cl1, Ccr5, Fas, Il13ra1, Ccl21, Il18r1, Csf1r, Il17re
rno04640	Haematopoietic cell lineage	7.99E−03	Il6r, Il9r, Mme, Csf3, Kitlg, Csf1r
rno04151	PI3K–Akt signalling pathway	2.78E−05	Hgf, Il6r, Fgfr2, Pdgfa, Pdgfra, Jak3, Spp1, F2r, Irs1, Csf3, Itgb7, Tlr4, Kitlg, Gng11, Prkaa2, Gng10, Csf1r, Epha2, Creb5
rno04014	Ras signalling pathway	5.39E−03	Hgf, Fgfr2, Pld1, Pdgfa, Pdgfra, Pla2g5, Kitlg, Gng11, Gng10, Csf1r, Epha2
rno05205	Proteoglycans in cancer	6.75E−04	Hgf, Casp3, Rock2, Tlr4, Fzd1, Tgfb2, Wnt2b, Wnt11, Fas, Wnt9b, Pxn, Mapk11
rno04550	Signalling pathways regulating the pluripotency of stem cells	1.51E−03	Hnf1a, Fgfr2, Jak3, Fzd1, Wnt2b, Wnt11, Wnt9b, Meis1, Mapk11
rno04916	Melanogenesis	3.11E−03	Edn1, Mitf, Fzd1, Kitlg, Wnt2b, Wnt11, Wnt9b

### 3.5 Construction of the PPI network and identification of hub genes

**FIGURE 7:**
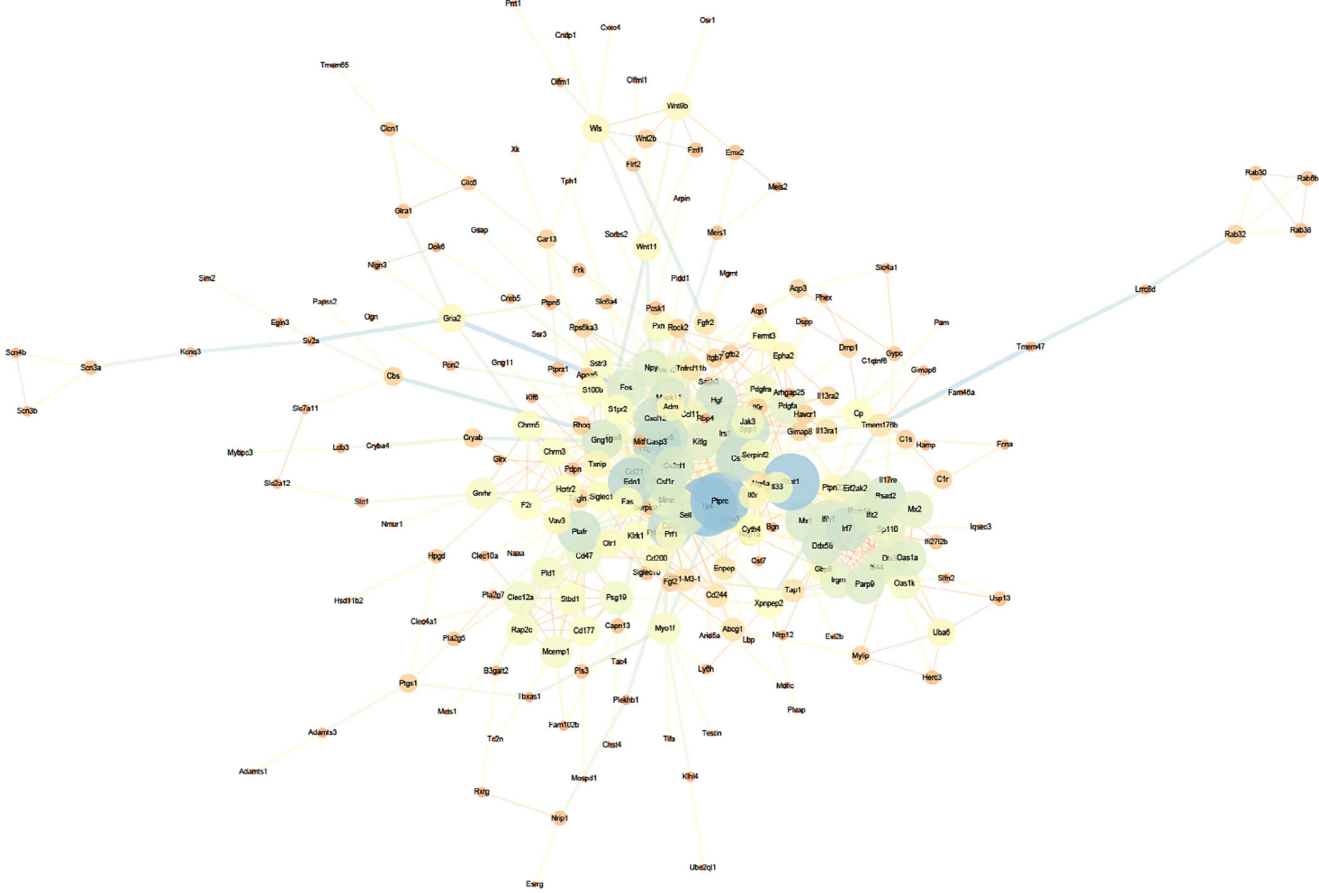
Protein–protein interaction network incorporating targeted genes

**FIGURE 8:**
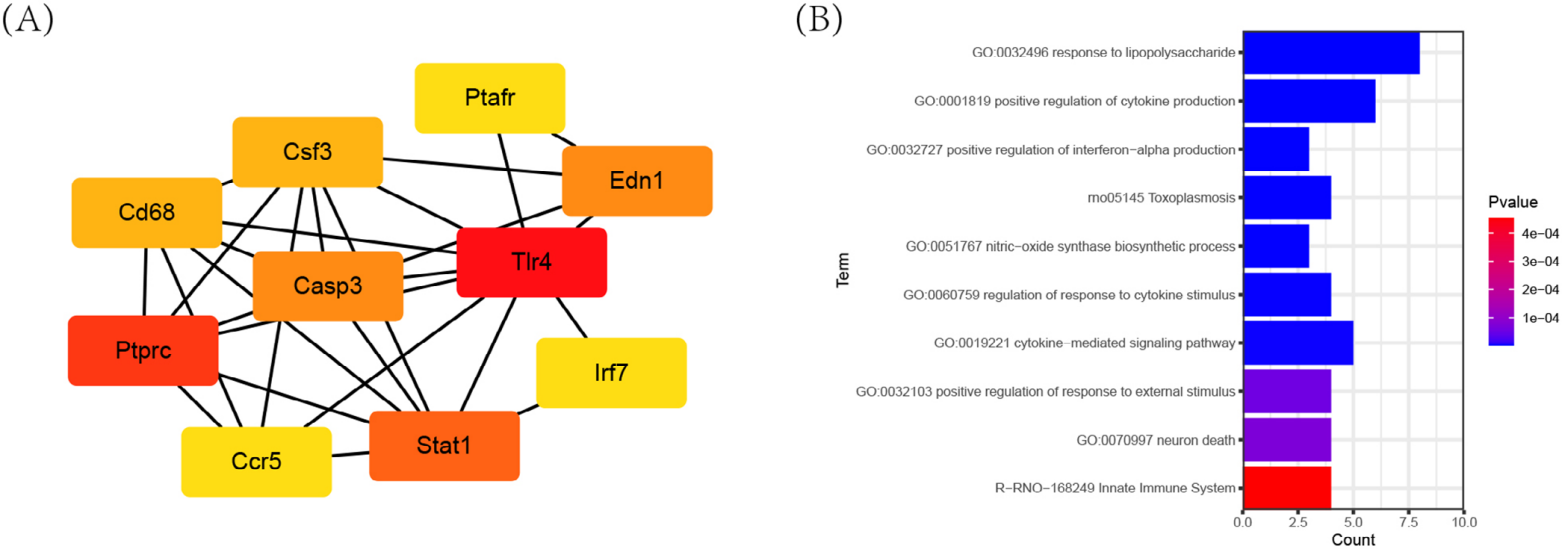
Functional enrichment analysis of the 10 hub genes. (A) Top 10 genes with the highest association in the PPI network by the cytoHubba plug‐in. (B) Functional enrichment analysis and pathways significantly related to hub genes. PPI, protein–protein interaction

As shown in Figure 7, we predicted a PPI network to display the interactions of target genes through the STRING database. Then, we recognized the top 10 genes with the highest association in the PPI network by the cytoHubba plug‐in (Figure 8A and Table 6). Functional enrichment analysis by Metascape indicated that eight GO terms, including reaction to lipopolysaccharide (GO:0032496), nitric oxide synthase biosynthetic process (GO:0051767), control of the response to cytokine stimulus (GO:0060759), cytokine‐mediated signalling pathway (GO:0019221), neuron death (GO:0070997), positive control of cytokine production (GO:0001819), interferon‐alpha production (GO:0032727) and response to external stimulus (GO:0032103), and two pathways, including toxoplasmosis (rno05145) and innate immune system (R‐RNO‐168249), were significantly related to hub genes (Figure 8B and Table 7).

**TABLE 6:** Top 10 genes with the highest degrees by CytoHubba

Rank	Name	Full name	Ensembl ID	Score
1	Tlr4	Toll‐like receptor 4	ENSRNOG00000010522	41
2	Ptprc	Protein tyrosine phosphatase, receptor type, C	ENSRNOG00000000655	40
3	Stat1	Signal transducer and activator of transcription 1	ENSRNOG00000014079	38
4	Casp3	Caspase 3	ENSRNOG00000010475	28
5	Edn1	Endothelin 1	ENSRNOG00000014361	28
6	Csf3	Colony stimulating factor 3	ENSRNOG00000008525	25
7	Cd68	Cd68 molecule	ENSRNOG00000037563	25
8	Irf7	Interferon regulatory factor 7	ENSRNOG00000017414	22
9	Ptafr	Platelet‐activating factor receptor	ENSRNOG00000013231	22
10	Ccr5	C‐C motif chemokine receptor 5	ENSRNOG00000049115	22

**TABLE 7:** Functional enrichment analysis of the 10 hub genes

Category	Term	Description	*p* Value	Symbols
GO biological processes	GO:0032496	Response to lipopolysaccharide	1.87368E−11	Edn1, Stat1, Casp3, Csf3, Tlr4, Ptafr, Ccr5, Cd68
GO biological processes	GO:0001819	Positive regulation of cytokine production	5.15929E−08	Ptprc, Stat1, Tlr4, Ptafr, Ccr5, Irf7, Casp3, Cd68
GO biological processes	GO:0032727	Positive regulation of interferon‐alpha production	2.21026E−07	Stat1, Tlr4, Irf7, Casp3, Ccr5, Ptprc, Edn1, Cd68
GO biological processes	GO:0051767	Nitric oxide synthase biosynthetic process	4.08255E−07	Edn1, Stat1, Tlr4, Ptafr, Casp3, Csf3
GO biological processes	GO:0060759	Regulation of response to cytokine stimulus	4.60603E−07	Edn1, Ptprc, Tlr4, Irf7, Casp3, Ptafr, Ccr5
GO biological processes	GO:0019221	Cytokine‐mediated signalling pathway	1.1734E−06	Edn1, Ptprc, Stat1, Ccr5, Irf7, Csf3, Ptafr, Cd68
GO biological processes	GO:0032103	Positive regulation of the response to external stimulus	5.30195E−05	Edn1, Tlr4, Ccr5, Irf7, Casp3
GO biological processes	GO:0070997	Neuron death	7.02504E−05	Casp3, Csf3, Tlr4, Ccr5, Ptprc, Stat1
KEGG pathway	rno05145	Toxoplasmosis	2.75493E−07	Stat1, Casp3, Tlr4, Ccr5, Csf3
Reactome gene sets	R‐RNO‐168249	Innate immune system	0.000453605	Ptprc, Tlr4, Ptafr, Cd68, Casp3

### 3.6 Validation of DE lncRNAs by RT‐PCR

**FIGURE 9:**
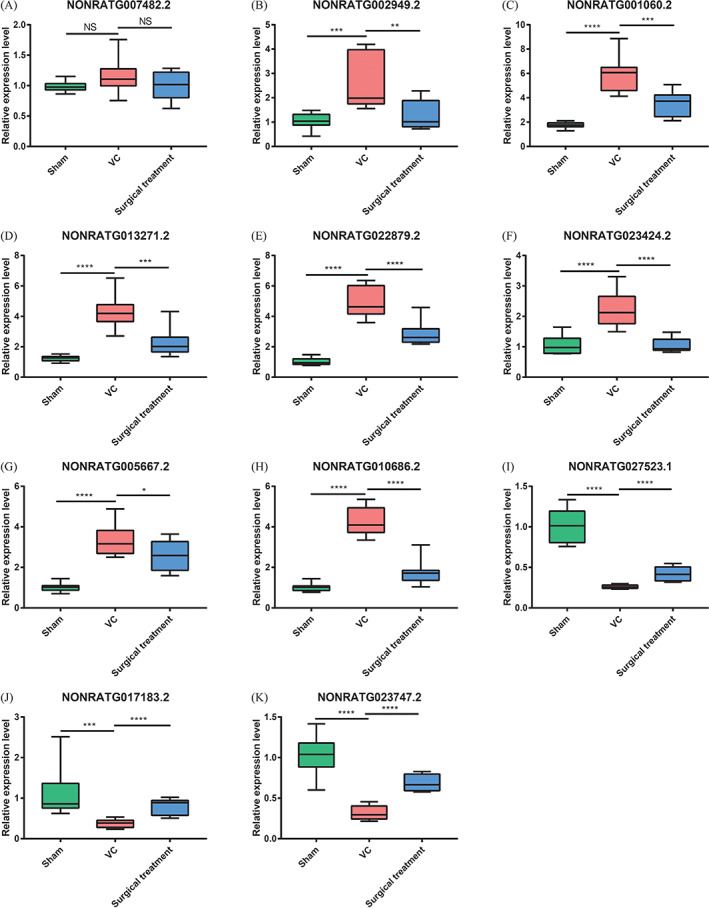
Validation of DE lncRNAs by RT‐PCR. *p < 0.05; **p < 0.01; ***p < 0.001; ****p < 0.0001. DE lncRNAs, differentially expressed lncRNAs; NS, not significant; RT‐PCR, quantitative real‐time polymerase chain reaction; Sham, sham group; Surgical treatment, surgical treatment group; VC, varicocele group

Furthermore, we verified the expression of DE lncRNAs in testicular tissues of VC rats by RT‐PCR. As illustrated in Figure 9, the relative expression of *NONRATG002949.2*, *NONRATG001060.2*, *NONRATG013271.2*, *NONRATG022879.2*, *NONRATG023424.2*, *NONRATG005667.2* and *NONRATG010686.2* was higher in the VC group than in the other two groups (*p* < 0.05). The relative expression of *NONRATG027523.1*, *NONRATG017183.2* and *NONRATG023747.2* in the VC group was downregulated significantly (*p* < 0.05). No difference in *NONRATG007482.2* expression was found (*p* > 0.05).

### 3.7 Correlation between DE lncRNA expression and sperm quality

**FIGURE 10:**
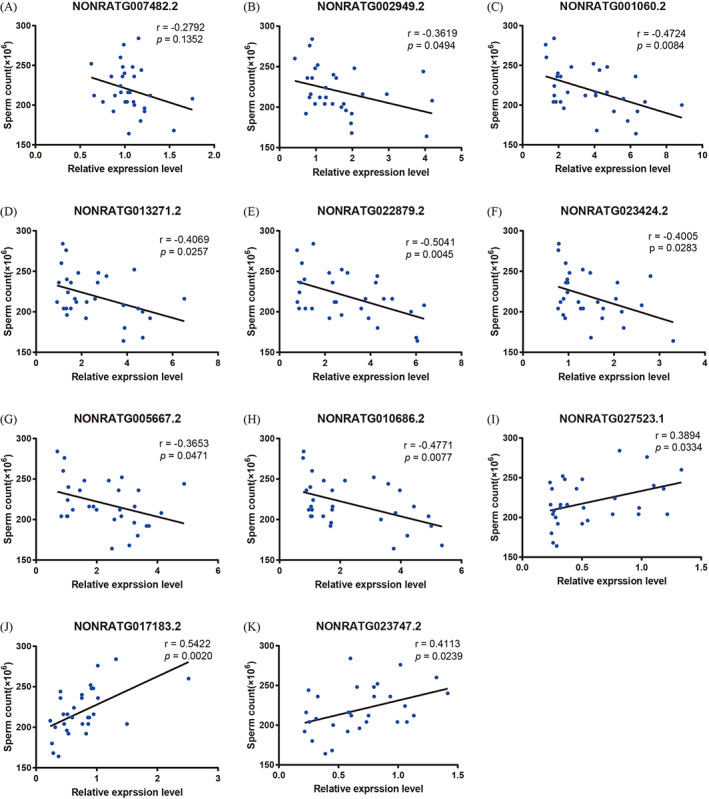
Correlation between DE lncRNA expression and sperm count. DE lncRNAs, differentially expressed lncRNAs

**FIGURE 11:**
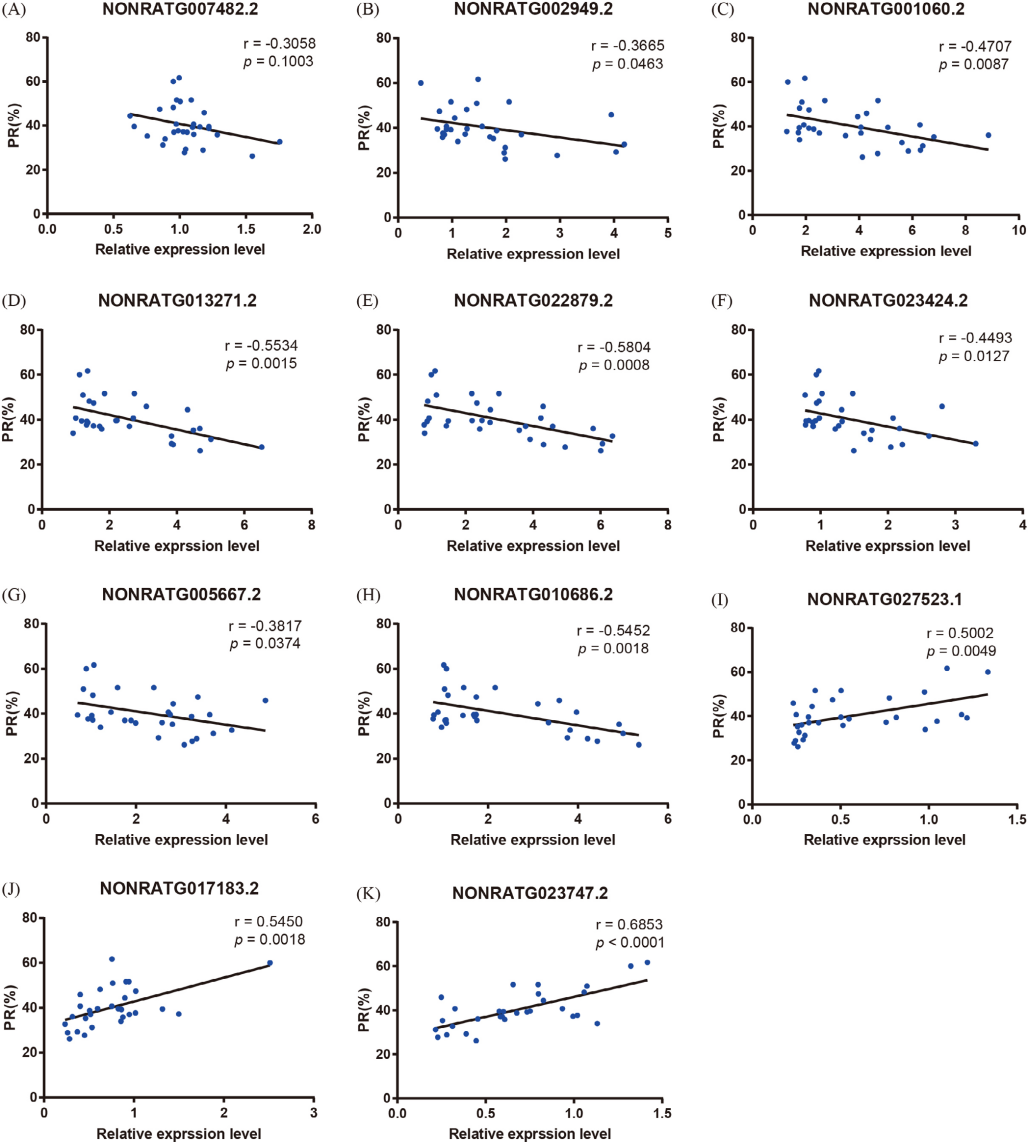
Correlation between DE lncRNA expression and the percentage of PR. DE lncRNAs, differentially expressed lncRNAs; PR, sperm progressive motility

To illustrate the effect of DE lncRNAs on the semen quality of VC rats, we performed a correlation analysis between the DE lncRNA expression and sperm count and motility. As shown in Figures 10 and 11, the relative expression of *NONRATG002949.2*, *NONRATG001060.2*, *NONRATG013271.2*, *NONRATG022879.2*, *NONRATG023424.2*, *NONRATG005667.2* and *NONRATG010686.2* was significantly negatively related to sperm count and the percentage of PR, while *NONRATG027523.1*, *NONRATG017183.2* and *NONRATG023747.2* showed the opposite trend (*p* < 0.05). No apparent association was exhibited between *NONRATG007482.2* expression and sperm quality (*p* > 0.05).

### 3.8 Validation of regulated spermatogenic cell apoptosis by DE lncRNAs by TUNEL staining of rat testicular tissues

**FIGURE 12:**
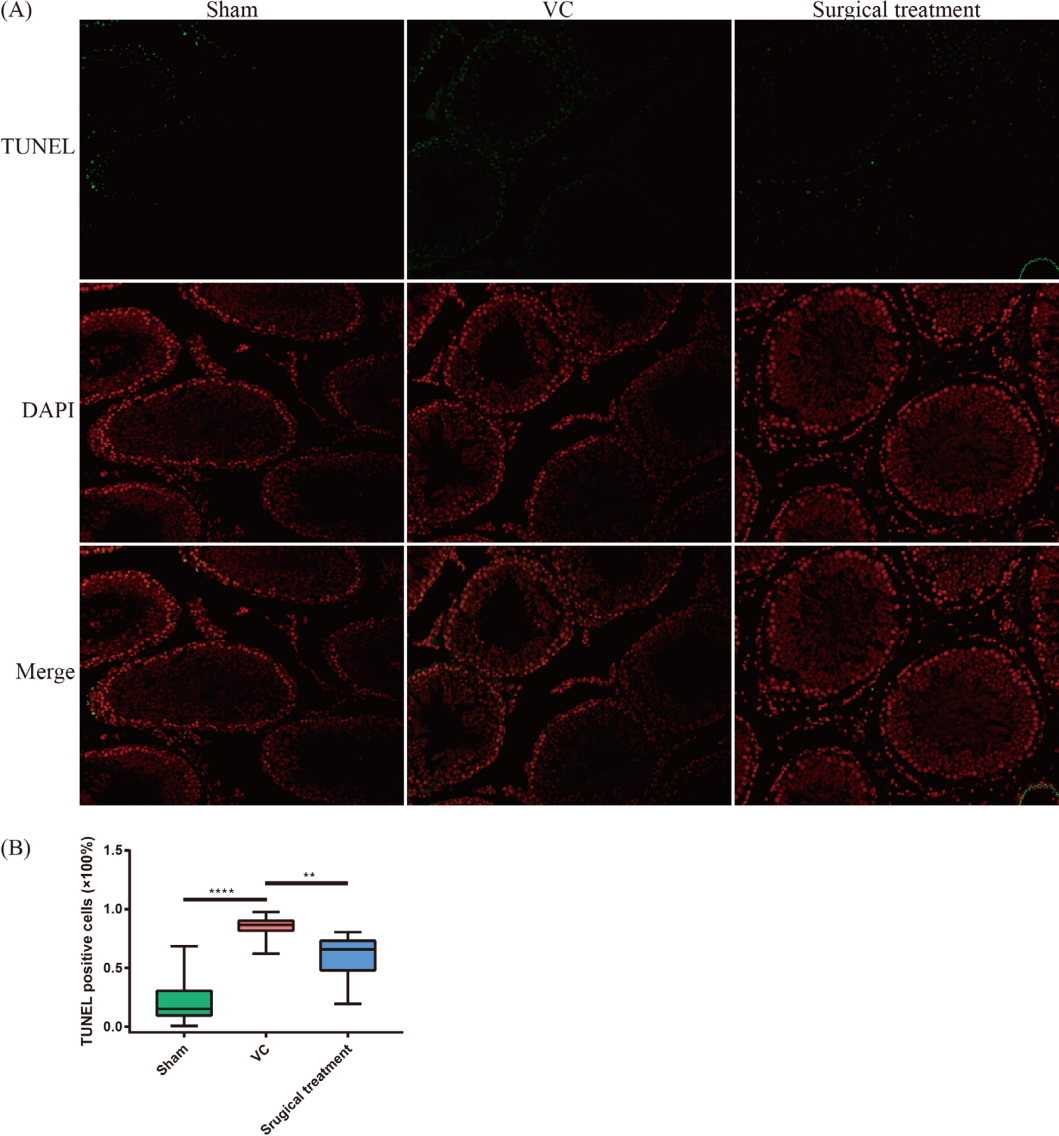
Spermatogenic cell apoptosis tests by TUNEL staining. (A) Representative TUNEL staining image of rat testicular tissues (×200). (B) The percentage of TUNEL‐positive cells. **p < 0.01; ****p < 0.0001. DAPI, 4′6‐diamidino‐2‐phenylindole; Sham, sham group; Surgical treatment, surgical treatment group; TUNEL, terminal deoxynucleotidyl transferase nick end labeling; VC, varicocele group

We performed TUNEL staining of rat testis tissue to investigate the regulation of DE lncRNAs on spermatogenic cell apoptosis. The TUNEL assay results showed that the percentage of apoptotic cells in the testicular tissue of VC rats increased but decreased after surgical intervention (Figure 12A,B). The DE lncRNAs regulate spermatogenic cell apoptosis in VC, and surgical treatment significantly improves apoptosis.

### 3.9 Validation of regulated signalling pathways, spermatogenic cell apoptosis and proliferation, and meiotic spermatocytes of DE lncRNAs by Western blot

**FIGURE 13:**
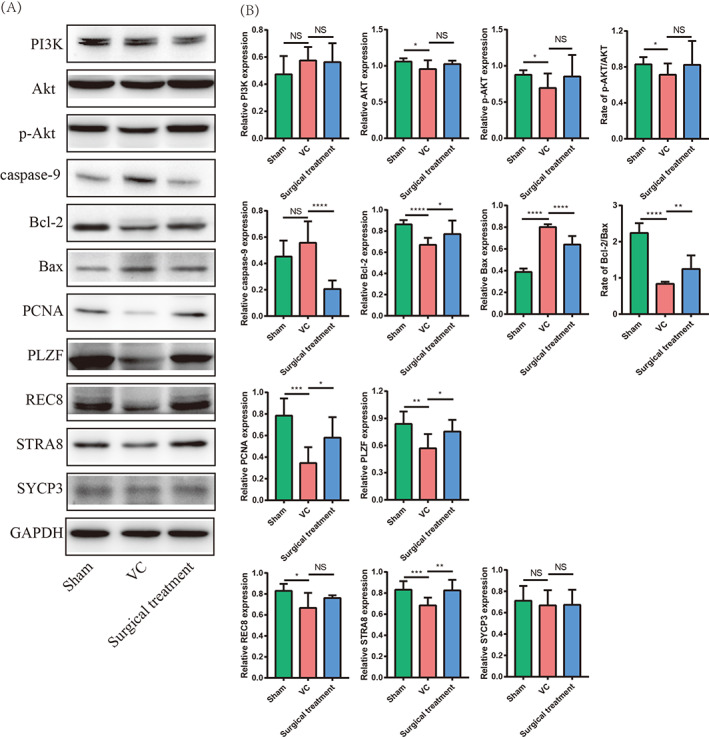
Validation of regulated signalling pathways, spermatogenic cell apoptosis and proliferation, and meiotic spermatocytes by Western blot. Representative Western blot images of PI3K, Akt, p‐Akt, caspase‐9, Bcl‐2, Bax, PCNA, PLZF, REC8, STRA8, and SYCP3. (B) Statistical analysis of band intensity by Student's *t*‐test. *p < 0.05; **p < 0.01; ***p < 0.001; ****p < 0.0001. NS, not significant; Sham, sham group; Surgical treatment, surgical treatment group; VC, varicocele group

**FIGURE 14:**
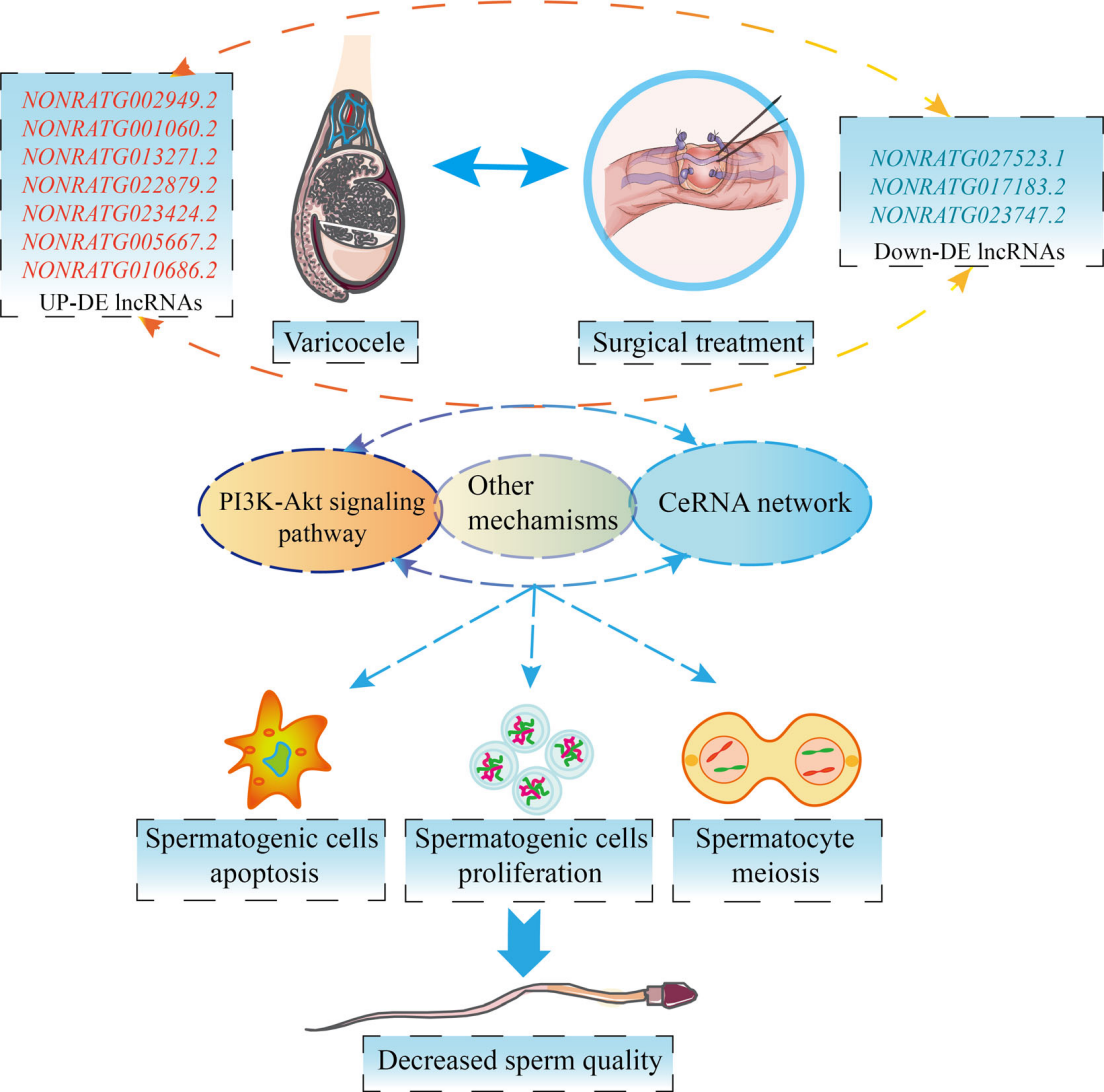
Long noncoding RNAs are regulated spermatogenesis in varicocele‐induced spermatogenic dysfunction. LncRNAs promote spermatogenic cell apoptosis and inhibit spermatogonia, spermatocyte proliferation, and meiotic spermatocytes by regulating the PI3K–Akt signalling pathway and other mechanisms

To validate the downstream signalling pathways and phenotypes, according to the DE lncRNAs functional enrichment and KEGG analyses (Figure 6), we selected the PI3k–Akt signalling pathway to validate by Western blot. More and more studies have shown that the PI3k–Akt signalling pathway regulated cell apoptosis, proliferation and spermatocytes in spermatogenesis. According to the DE lncRNA functional enrichment, TUNEL, and HE staining results, we selected the phenotypes of spermatogenic cell apoptosis, spermatogenic cell proliferation and meiotic spermatocytes to validate by Western blot. The results are shown in Figure 13. We found that the expression of PI3K did not change in the three groups (*p* > 0.05), and the expression of Akt and p‐Akt was decreased in the VC group compared with the sham group (*p* < 0.05) and partly restored in the surgical treatment group (*p* > 0.05). The expression of caspase‐9 and Bax was increased and Bcl‐2 was decreased in the VC group compared with the sham group and restored after surgical treatment (*p* < 0.05). The expression of PCNA and PLZF was decreased in the VC group compared with the sham group and restored after surgical treatment (*p* < 0.05). The expression of REC8 and STRA8 was decreased in the VC group compared with the sham group. STRA8 expression was restored after surgical treatment (*p* < 0.05), but REC8 showed no significant changes after surgical treatment (*p* > 0.05). The expression of SYCP3 did not change in the three groups (*p* > 0.05). These results suggested that DE lncRNAs promote spermatogenic cell apoptosis and inhibit spermatogonia and spermatocyte proliferation and spermatocyte meiosis by regulating the PI3K–Akt signalling pathway (Figure 14).

### 3.10 Identification of the coexpression of DE lncRNAs in the sham group

Compared with lncRNAs in the sham group, 244 upregulated lncRNAs and 27 downregulated DE lncRNAs were detected in the VC group (Figure S1A,C). In the comparison between the sham group and the surgical treatment group, we identified 51 DE lncRNAs, including 42 upregulated and 9 downregulated DE lncRNAs (Figure S1B,D). Venn diagram analysis showed that 19 DE lncRNAs overlapped between the two comparisons (Figure S1E,F). Specifically, all the 19 DE lncRNAs were upregulated and no DE lncRNAs were downregulated in the sham group.

## 4 DISCUSSION

VC is a common risk factor for male infertility.^1^ However, some people with VC have infertility or negative sperm quality, whereas others do not. The connection between VC and spermatogenic dysfunction is still controversial. LncRNAs play various functions in many diseases through signalling pathways.^22^, ^23^ Previous studies have shown that lncRNAs play roles in VC‐related male infertility through the regulation of hypoxia responses.^23^ Although a large number of lncRNAs have been found in the testis, their biological function remains to be further studied in various diseases. There are few studies on lncRNA expression patterns in VC‐induced spermatogenic dysfunction. Thus, how ncRNAs are expressed during spermatogenesis before and after VC surgical repair must be further understood.

In the present study, lncRNA sequencing technologies were used to analyse lncRNA expression in rats with VC. Our results showed that lncRNA expression was different in the VC group compared with the sham group or surgical treatment group. We considered the coexpression DE lncRNAs in VC group would be the key lncRNAs in VC‐induced spermatogenic dysfunction. So, we analysis and validated the functions of the eight lncRNAs were significantly coincreased and three lncRNAs were significantly codecreased in the VC group versus sham group and surgical treatment group.

We validated the expression of the 11 lncRNAs in the three groups using RT‐PCR. The results were in accord with the RNA‐seq data analysis, except for lncRNA NONRATG007482.2. Then, we constructed a ceRNA network and suggested that 4 lncRNAs potentially interact with 2 miRNAs (miR‐301a‐5p and miR‐328a‐5p) and 12 mRNAs, and we conducted functional enrichment analysis of target genes and KEGG analyses. These biological processes are primarily involved in inflammation, oxidative stress and cell apoptosis, and so on. Depending on functional enrichment and KEGG analyses, we selected PI3k–Akt signalling pathway and the phenotypes of spermatogenic cell apoptosis, spermatogenic cell proliferation and meiotic spermatocytes to validate by Western blot. And the results showed that DE lncRNAs promote spermatogenic cell apoptosis, and inhibit spermatogenic cell proliferation and meiotic spermatocytes by regulating the PI3K–Akt signalling pathway and other mechanisms.

LncRNAs are important mediators of the ceRNA regulatory network, and they can absorb miRNAs and regulate the expression of target genes.^24^ We constructed a ceRNA network and suggested that 4 lncRNAs potentially interact with 2 miRNAs (miR‐301a‐5p and miR‐328a‐5p) and 12 mRNAs. We identified more VC‐induced spermatogenic dysfunction and surgical repair‐specific ceRNA pairs than previous studies. In previous studies, miRNAs as biomarkers for VC have been widely discussed. Xu et al. analysed the expression of miR‐210‐3p in patients with VC and found that the level of miR‐210‐3p in the seminal plasma of patients with VC was 2.18 times higher than that of healthy people.^25^ Zhi et al. found that miR‐192a could be a predictive factor for the spermatogenic status of patients after VC repair.^26^

However, studies of the functions of miR‐301a‐5p and miR‐328a‐5p in VC or spermatogenesis are still lacking. Our study may provide two potential diagnostic and therapeutic candidates for VC‐induced spermatogenic dysfunction. miR‐301a‐5p and miR‐328a‐5p participate in biological processes in many other diseases. Wang et al. found that lncRNA EPB41L4A‐AS2 sponges miR‐301a‐5p and inhibits hepatocellular carcinoma development.^27^ The expression of miR‐301a‐5p was certified in gastric cancer tissues, and high miR‐301‐5p expression was found to be associated with the aggressiveness of gastric cancer.^28^ Huo et al. revealed that downregulated lncRNA‐MIAT could activate miR‐328a‐5p against erectile dysfunction in diabetes mellitus rats.^29^ Through animal experiments, miR‐328a‐5p was found to be downregulated in a rat model of acute kidney injury induced by contrast agents.^30^

Through functional enrichment analysis of target genes and KEGG analyses, we identified several signalling pathways and biological processes related to these lncRNAs. Our results suggested that DE lncRNAs were predominantly enriched in ‘immune effector process,’ ‘regulation of immune effector process,’ ‘leukocyte apoptotic process’ and ‘response to oxidative stress.’ Most DE lncRNAs participated in ‘pathways in cancer,’ ‘PI3K–Akt signalling pathway’ and ‘Ras signalling pathway.’ These biological processes are primarily involved in inflammation, oxidative stress and cell apoptosis.

Many studies have shown that inflammatory mechanisms play important roles in VC.^31^, ^32^ IL‐1α and IL‐1β have been reported to be increased in the VC model.^33^, ^34^ Camargo et al. found that IL‐1, IL‐18 and caspase‐1 decreased in semen after varicocelectomy by ELISAs.^35^ Micheli et al. tested seminal plasma samples and found that sperm apoptosis, IL‐6, and TNF‐α were increased in VC patients.^36^ Zeinali et al. measured IL expression in 75 infertile men with VC and showed that IL‐18 increased and activated neutrophils and oxygen species in infertile patients with VC.^37^ These available studies and our present study data support that inflammation may play an essential role in the progression of spermatogenic dysfunction in VC.

We also found that lncRNAs and miRNAs are involved in the adjustment of oxidative stress, which plays an important role in VC‐associated spermatogenic dysfunction. The relationship between oxidative stress and sperm damage in VC patients has been investigated by Ammar et al., who found that impaired seminal antioxidant capacity and elevated seminal levels of lipid peroxidation may contribute to the aetiology of nuclear sperm DNA damage in VC patients.^6^ Ata‐Abadi et al. used RNA sequencing datasets from Gene Expression Monibus to identify hypoxia‐responding lncRNAs, evaluated the expression of lncRNAs by RT‐PCR and analysed their expression in patients with VC.^38^ The results showed that these lncRNAs, including MIR210HG and MLLT4‐AS1, were positively correlated with oxidative stress and negatively correlated with sperm quality in men with VC. Oxidative stress‐related expression patterns of miR‐21, miR‐34a and miR‐122a were found to be decreased among patients with severe VC, particularly those with defective spermatogenesis in Ashrafzade et al.'s study.^39^

The DE lncRNAs in our present study were not reported in a previous study of VC. These lncRNAs may be potential novel biomarkers for predicting the risk of spermatogenic dysfunction in VC and the effect of surgical repair. However, we randomly selected three rats from each group for lncRNA sequencing, the sample size for RNA‐seq was small. The sample‐to‐sample variability may affect the stability of our study. Therefore, we further validated the expression of DE lncRNAs by RT‐PCR and evaluated the correlation of the expression of the key lncRNAs with sperm quality. We found that a total of seven lncRNAs were negatively correlated with total sperm count and sperm PR motility, while three lncRNAs were positively correlated. The lncRNAs as biomarkers for predicting the risk of spermatogenic dysfunction in VC have been reported in previous studies. A study indicated that lncRNAs SLC‐AS6 and SLC‐AS7 were negatively correlated with sperm count and motility in male infertile sperm samples associated with VC.^15^ Zhao et al. found that lncRNA gadd7 could promote the apoptosis of mouse spermatocytes in mice with VC‐induced infertility.^15^

To validate the downstream signalling pathways and phenotypes, according to the KEGG analyses, we selected the PI3k–Akt signalling pathway to validate by Western blot. We found that the expression of p‐Akt protein decreased in the testes of VC rats. Previous studies showed the same results as our study of PI3K–Akt signalling pathway regulated spermatogenesis.^40^, ^41^, ^42^ Aquila et al. study found that estradiol could enhance phosphorylation of the protein Akt, which was regarded as a germ cell survival factor in the human testis.^43^ Dube et al. tested the epididymis tissues by RT‐PCR, and found that decreased expression of epidermal growth factor increasing PI3K–Akt signalling pathway regulated the specific luminal microenvironment necessary for the creation of fertilizing‐competent spermatozoa.^44^ Wang et al.^45^ used a VC rat model study and found that the PI3K–Akt signalling pathway plays a regulatory role in VC‐induced spermatogenesis disorder. Zhao et al.^40^ performed in vitro experiments and found that the PI3K–Akt signalling pathway participates in regulating sprmatogonial cell apoptosis and proliferation. A previous study indicated that lncRNAs also induce spermatogenic cell apoptosis in VC patients.

According to DE lncRNA functional enrichment, TUNEL and HE staining results, we selected the phenotypes of spermatogenic cell apoptosis, spermatogenic cell proliferation and meiotic spermatocytes to validate by Western blot. We found that the expression of the apoptosis‐promoting proteins caspase‐9 and Bax was increased, and the apoptosis‐inhibiting protein Bcl‐2 was decreased in the testes of VC rats. The TUNEL assay results indicated that the percentage of apoptotic spermatogenic cells in the testis tissue of VC rats increased and decreased after surgical intervention. Some apoptotic mechanisms were believed to be connected with VC, originating in the mitochondria of spermatocytes and working in the nucleus in Wu et al.'s study.^9^ Zhao et al. found that lncRNA gadd7 was upregulated in the semen of VC patients, and an in vitro study indicated that overexpression of lncRNA gadd7 induced the apoptosis of spermatocytes and suppressed GC‐1 and GC‐2 cell proliferation.^46^, ^47^

We detected the expression of PCNA and PLZF, two biomarkers associated with spermatogonia and spermatocyte proliferation and differentiation.^48^, ^49^ We found that the expression of PCNA and PLZF was decreased in VC rats and restored after surgical repair. STRA8, REC8 and SYCP3 are biomarkers associated with meiotic spermatocytes.^49^, ^50^ Our results showed that the expression of STRA8 and REC8 was decreased in VC rats. STRA8 is a biomarker for entry of germ cells into meiotic prophase I, and REC8 is a meiotic marker gene.^49^, ^50^ These results indicated that DE lncRNAs promote spermatogenic cell apoptosis and inhibit spermatogonia and spermatocyte proliferation and meiotic spermatocytes via the PI3K–Akt signalling pathway, which may affect subsequent spermatogenesis processes and sperm quality in VC.

Our study provides a foundation for the expression signature of lncRNAs and preliminarily studies the functions and mechanisms of lncRNAs regulating the process of spermatogenesis in VC‐induced spermatogenic dysfunction and surgical repair. Our study has some limitations. First, the sample size for lncRNA‐seq was small. Second, nosogenesis may vary in different VC periods. Third, the functions of lncRNAs were predicted from bioinformatics analyses. Spermatogenesis is a complex biological process, and the functions of lncRNAs at different stages of VC require more systemic investigations. Therefore, more studies based on larger sample sizes and in different stages of VC are necessary in the future.

## 5 CONCLUSION

Our study provides a foundation for the expression signature of lncRNAs to understand the molecular mechanisms in VC‐induced spermatogenic dysfunction and surgical repair. Ten DE lncRNAs were associated with sperm quality in VC. The 10 DE lncRNAs may be potential novel biomarkers for predicting the risk of spermatogenic dysfunction in VC and the effect of surgical repair. These DE lncRNAs promote spermatogenic cell apoptosis and inhibit spermatogonia and spermatocyte proliferation and meiotic spermatocytes by regulating the PI3K–Akt signalling pathway. The functions of lncRNAs in VC‐induced spermatogenic dysfunction require more systemic investigations in the future.

## CONFLICTS OF INTEREST

The authors declare no conflicts of interest.

## AUTHOR CONTRIBUTIONS

Xiaoqiang Liu conceived the project. Shangren Wang, Jiaqi Kang, Yuxuan Song, Aiqiao Zhang and Yang Pan performed the experiment and wrote the manuscript. Zhexin Zhang and Yuezheng Li analysed data. Li Liu, Shuai Niu and Xiaoqiang Liu provided the resources. All authors read and approved the final manuscript.

## Supporting information


**FIGURE S1** Identification of the coexpression of DE lncRNAs in the sham group

## Data Availability

The data that support the findings of this study are available from the corresponding author upon reasonable request.
