# Supplementary figures and images for: Long noncoding RNAs regulated spermatogenesis in varicocele‐induced spermatogenic dysfunction
Source: Cell Prolif. 2022 Mar 17;55(5):e13220. doi: 10.1111/cpr.13220 (PMC9136499; doi:10.1111/cpr.13220)

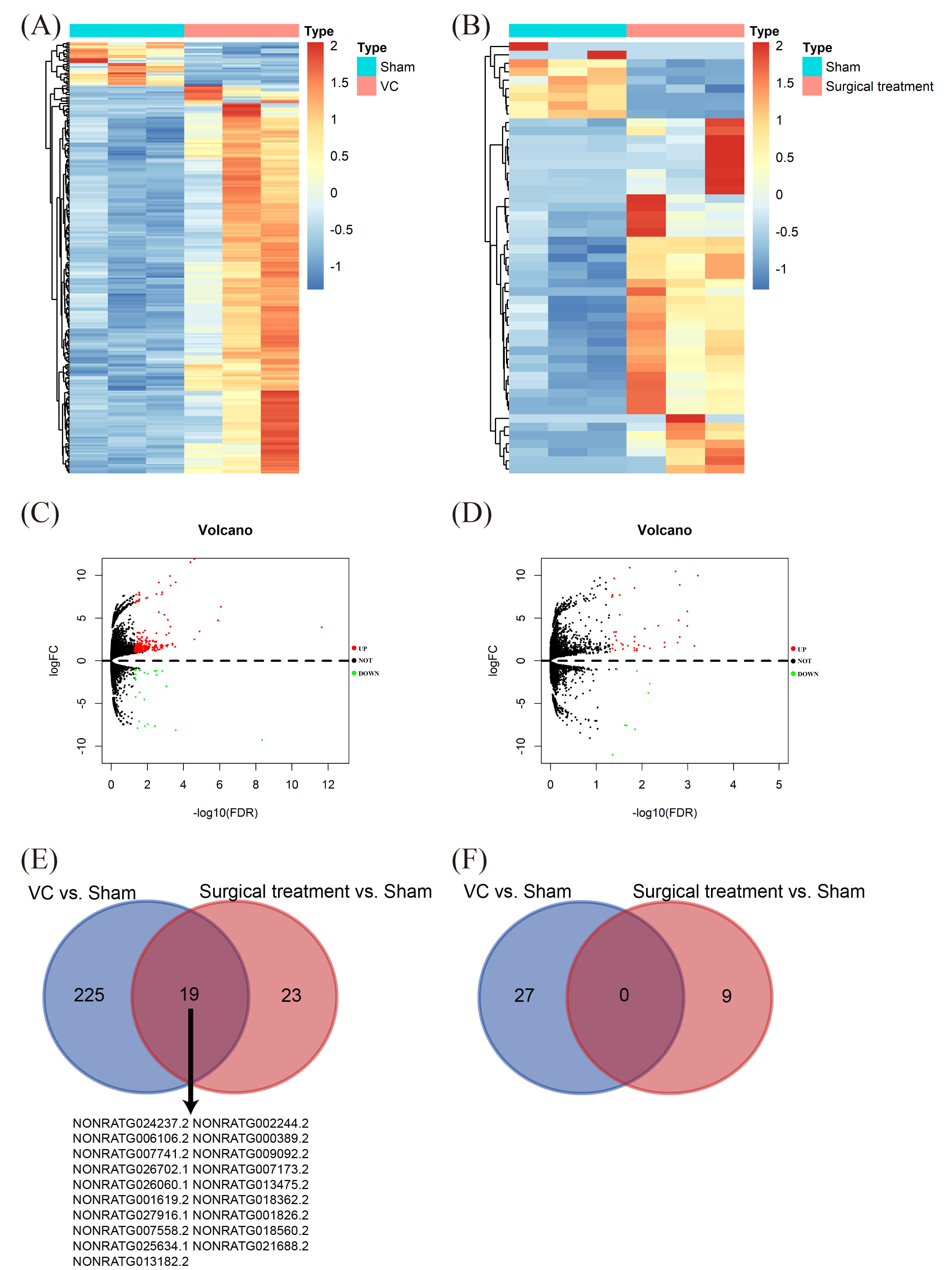

Supplement: Supplementary file 1 — FIGURE S1 Identification of the coexpression of DE lncRNAs in the sham group [file CPR-55-e13220-s001.jpg]
